# Identification and Expression Analysis of *Wnt2* Gene in the Sex Differentiation of the Chinese Soft-Shelled Turtle (*Pelodiscus sinensis*)

**DOI:** 10.3390/life13010188

**Published:** 2023-01-09

**Authors:** Tong Zhou, Haiqi Zhang, Meng Chen, Yingping Zhang, Guobin Chen, Guiwei Zou, Hongwei Liang

**Affiliations:** 1 Yangtze River Fisheries Research Institute, Chinese Academy of Fishery Sciences, Wuhan 430223, China; 2 Zhejiang Institute of Freshwater Fisheries, Huzhou 313001, China; 3 Key Laboratory of Aquatic Genomics, Ministry of Agriculture and Rural Affairs, Yangtze River Fisheries Research Institute, Chinese Academy of Fishery Sciences, Wuhan 430223, China

**Keywords:** *Pelodiscus sinensis*, *Wnt2*, gonad differentiation, Wnt agonist

## Abstract

The Chinese soft-shelled turtle (*Pelodiscus sinensis*) is an important freshwater aquaculture animal in China. The *Wnt* gene family plays important regulatory roles in the development and growth of mammals. However, the precise function of these family genes has not been well understood in the sex differentiation of Chinese soft-shelled turtles. Here, we cloned a member of the Wnt family, *Wnt2*, which obtained a 1077 bp open reading frame that encoded a 358-aa protein. The putative amino acid sequences of proteins are exceeded 80% identical to other turtles. The expression level of *Wnt2* peaked at the 14th stage both in female and male embryos during the early gonadal differentiation period of Chinese soft-shelled turtles, which occurred before gonadal differentiation. *Wnt2* mRNA was expressed at higher levels in the brains and gonads of mature *P. sinensis* females compared with those in mature males. Wnt agonists significantly affected the expression level of *Wnt2* during the gonadal differentiation period. After Wnt agonists (1.0 μg/μL, 2.5 μg/μL, 5.0 μg/μL) treatment, the expression level of the *Wnt2* generally appeared to have an inverted-V trend over time in female embryonic gonads. The results suggested that *Wnt2* may participate in the regulation of gonad development in *P. sinensis* during the early embryonic stages. These results could provide a theoretical basis for the reproduction process of the Chinese soft-shelled turtle.

## 1. Introduction

*Pelodiscus sinensis*, a Chinese soft-shelled turtle, is one of China’s economically important freshwater aquaculture animals [1]. When exposed to an appropriate temperature ranging from 24 to 34 °C, the *P. sinensis* eggs could develop normally [2]. Within this range, the temperature may not affect the sex ratio and hatching success of hatchling embryos. When eggs were incubated at 30 °C, the primitive gonads began to appear on the 15th day. The gonads undergo a process of growth and development, and the gonad differentiation is completed until the 26th day (20 stages) [3]. Unlike other animals, males are highly popular and have higher prices than females due to the fact that males are superior to females in weight, size, and calipash width, as well as lower fat content [4]. The previous study showed that the Chinese soft-shelled turtle was a genotypic sex determination mechanism having a ZZ/ZW sex determination system [5]. Therefore, it allows studying genetically all-male populations in this species.

The Wnt signaling pathway is involved in important life activities such as organ formation and early development through signal transduction regulated by Wnt genes. Many Wnt genes have been observed in many higher eukaryotes, ranging from humans to Xenopus and zebrafish [6,7,8,9]. Further, Wnt signaling genes related to turtles’ gonadal development and differentiation have been identified, such as Wnt4 and Rspo1 [10,11].

The role of Wnt signaling in several development processes has been suggested that several Wnt genes are expressed in different organisms. Previous studies showed that Wnt4-deficient could cause sex reversal and steroidogenic function alteration in female mice [12], whereas mice null for frizzled 4 (Fzd4) are found to infecundity and reveal the damage of the corpus luteum [13,14]. In contrast, it has been found that targeted disruption of the *Wnt2* gene caused placentation defects [15], while Wnt2-null female mice were fertile [16]. Approximately 50% of Wnt2 knockout mice died soon after birth, possibly due to respiratory failure [17]. Wnt2, Wnt3, Wnt5a, and Wnt11 are involved in both follicular genesis and oogenesis [18], whereas during spermatogenesis of rainbow trout, other Wnt genes, such as Wnt5a, Wnt6, and Wnt7b are expressed in the testis [19].

Wnt2, a gene coding a factor from the Wnt family of signaling molecules, mediates the canonical Wnt/beta-catenin signaling pathway. Recent evidence suggests that the Wnt2 has been implicated in the development and differentiation process in invertebrate and vertebrate animals. Most previous studies have focused on expression patterns and regulatory mechanisms of the Wnt2 gene, which has been reported in mice [20], Pacific white shrimp [21], sea urchin [22] and flatworms [23]. Wnt2 exhibit the dimorphic expression pattern between male and female in the somatic gonadal cells of *D. melanogaster*, which is related to the doublesex (dsx) gene, which is required for male and female sex differentiation [24]. Additionally, the importance of Wnt2 ligand expression in follicles development has been well documented. For example, Wnt2 expression has been detected in rat follicular granulosa cells and in human cumulus cells and granulosa cells [25,26,27,28]. Wnt2 is reported to be highly expressed in *Hyriopsis cumingii* at all stages of ovarian development and 4 months of age gonads tissues, which suggests Wnt2 might be involved in the follicular formation and early gonadal development [29].

However, there is still no report focused on the role of Wnt2 in *P. sinensis*. Identification of Wnt2 expressed pattern in gonads tissues and embryos stage is essential for a broad understanding of their functions in gonadal development, potentially sex determination, and differentiation. In addition, we showed here information on the dynamics of *Wnt2* gene expression in developing gonads after the treatment of Wnt agonist could provide insight into the early development of both types of embryos. Therefore, this research could greatly widen our insights on *P. sinensis* Wnt2 and uncover their potential regulatory roles in the sex determination and reproduction processes of reptiles.

## 2. Materials and Methods

### 2.1. Sample Collection 

In this study, Chinese soft-shelled turtle fertilized eggs, three male and three female adults (mean weight 1000 g) were collected from Anhui Xijia Agricultural Development. Freshly laid fertilized eggs were collected and incubated in a constant temperature and humidity incubator. During the incubation process, the temperature was kept at 30 ± 0.5 °C, and humidity was controlled at 80–85%. Furthermore, embryos from stages 14–28 were sampled [30]. The details of sample collection have been portrayed previously [31]. Twenty embryo samples of different stages were processed, the ZZ/ZW-type sex of the embryos was identified based on sex-related markers developed [32], and then the embryos were used for quantitative expression analysis of embryonic gonadal development. 

Nine tissues, including the brain, liver, heart, spleen, muscle, kidney, lung, intestines, and gonads, were obtained from healthy adult turtles *P. Sinensis* after MS-222 anesthesia. Finally, they were quickly collected and frozen in liquid nitrogen, then stored at −80 °C for RNA extraction. The research was performed in accordance with provisions for the Yangtze River Fisheries Research Institute Animal Care Committee and the Guidelines for the Care and Use of Laboratory Animals.

### 2.2. Tissue Distribution of Wnt2 Transcripts

Total RNA was extracted from tissues, and then the first-strand cDNA was synthesized with PrimeScript™ RT reagent kit with gDNA Eraser (Takara, Dalian, China). The quantitative real-time PCR was conducted using the QuantStudio 5 Real-time PCR system (ABI, USA). RNA extraction, cDNA synthesis, and qRT-PCR were prepared from diverse tissues of *P. sinensis* under the same reaction conditions as mentioned in our study [31]. The relative expression of the *Wnt2* gene was determined by the comparative Ct (2^−ΔΔCt^) method using the 18S rRNA gene as the reference gene [33] (Table 1). 

### 2.3. Cloning of Full-Length cDNAs Encoding Wnt2

The PCR primers (Table 1) were designed to amplify the cDNA core fragment based on the mRNA sequences from NCBI data (Accession number: XM_006122543.3). Two fragments coding the core sequence of the *Wnt2* gene were amplified by RT-PCR with HiScript^®^ III 1st Strand cDNA Synthesis Kit and 2 × Rapid Taq Master Mix (Vazyme Bio, Inc., Nanjing, China). The reaction system was produced in a volume of 50 μL as follows: 25 μL 2 × Rapid Taq Master Mix, 1 μL cDNA, 1μL reverse primer (10 μM), 1 μL forward primer (10 μM), and 22 μL RNase-free ddH_2_O. The reaction conditions were as follows: pre-denaturation for 5 min at 95 °C; 95 °C for 30 s, 61 °C for 30 s, and 72 °C for 45 s, in 35 cycles; Extension at 72 °C for 10 min. Then PCR products were purified and sequenced in Wuhan Tianyihuiyuan Biotech Company.

The full-length cDNAs encoding Wnt2 from *P. Sinensis* were obtained using rapid amplification of cDNA ends (RACE). The 3′-RACE first-strand cDNAs were synthesized from 1 μg of total RNA from the ovary of *P. Sinensis* using the SMARTer^®^ RACE 5′/3′ Kit (Takara) according to the manufacturer’s instructions. Each 3′-RACE first-strand cDNA was used as a template, and amplification was primed by the Wnt2-3′-GSP and UPM short (Table 1). The gene-specific primers (Wnt2-3′-GSP, Table 1) were designed based on the partial sequence of obtained cDNA fragments. The PCR of Wnt2 3′-RACE was performed according to the following program: 94 °C for 5 min, 25 cycles of 94 °C for 30 s, 68 °C for 30 s, and 72 °C for 3 min. The PCR products of 3′-RACE were subcloned into the pMD18-T vector (Takara) and sequenced.

### 2.4. Sequence Alignment and Analysis

The open reading frame (ORF) and amino acid sequences were predicted with an online program (http://www.bio-soft.net/sms/index.html (accessed on 7 March 2022.)) and SMART (http://smart.embl-heidelberg.de). Twelve representative sequences for Wnt2 amino acid sequences were obtained with the NCBI database. Multiple sequence alignment of Wnt2 amino acid sequences was performed using DNAMAN software 8.0. The ExPASy Molecular Biology server (http://www.us.expasy.org/) and SignalP 3.0 Server (http://www.cbs.dtu.dk/services/-SignalP (accessed on 8 March 2022.)) were applied to perform the Sequence analysis. A phylogenetic tree was conducted using MEGA-X software 10.0 with the neighbor-joining method. 

### 2.5. Wnt Agonist Treatment

Wnt agonist (Selleck, Houston, TX, USA; NO. S8178) was used to mimic the effects of Wnt signaling at the whole organism level. Wnt agonist was identified as a small-molecule agonist of the Wnt signaling pathway. The activation of Wnt signaling also leads to the transcriptional activity of TCF/β-catenin and is independent of GSK-3β activity [34]. Wnt agonist treatments were carried out in accordance with the previous study [31]. 

Egg incubations were performed under the same condition as previously described. After 15 days of incubation, embryos were injected with different concentrations of Wnt agonist: 1.0 μg/µL, 2.5 μg/µL, and 5.0 μg/µL. The Wnt agonist was first prepared with ethanol as 10 μg/µL mother liquor and then diluted with a combination of 5% DMSO, 40% PEG 300, 5% Tween 80, and 50% ddH_2_O. The Control group was treated with 1μL of the mixture containing DSMO, PEG300, Tween 80, and ddH_2_O. At 0, 6, 12, 24, 36, 48, 72, 96, 120, 144, and 168 h time-point post injection (pi), 20 embryos of the experimental and control groups were collected. The type of the embryos was detected using sex-related markers, and the gonads of embryos were sampled for Wnt2 mRNA expression analysis. 

### 2.6. Statistical Analysis

We used SPSS software 17.0 (SPSS Inc, Chicago, IL, USA), one-way analysis of variance (ANOVA), and Duncan’s multiple comparison test was used to compare the data derived from different groups. Statistical evaluation of significance between different experimental groups was determined by a *p*-value of less than 0.05. Data were presented as mean ± S.D. (standard deviation of the mean; *n* = 9).

## 3. Results

### 3.1. Cloning and Sequence Analysis

**Figure 1:**
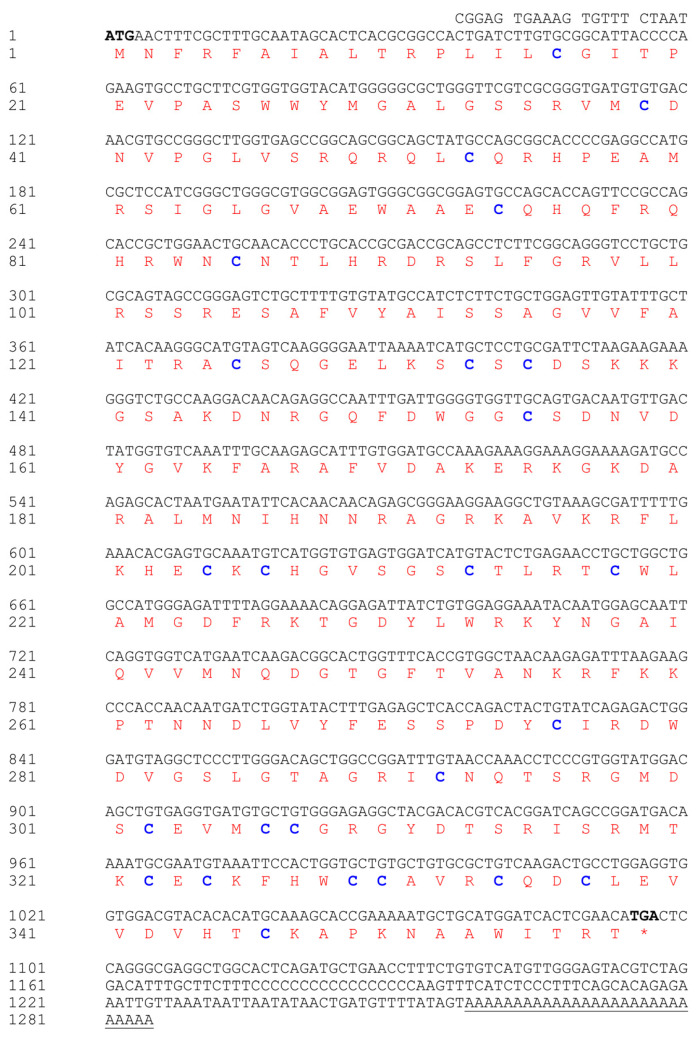
*Wnt2* cDNA sequence and putative amino acid of *P. sinensis.* The start codon and end codon are presented in bold black. The red denotes the WNT domain. The termination codon is shown with an asterisk, and the Poly A tail is marked with an underscore. “c” represents cysteines in the *Wnt2* amino acid sequence and is marked blue.

To explore the molecular functions of *Wnt2*, the cDNA sequence of *Wnt2* containing the entire coding region was cloned from the Chinese soft-shelled turtle *P. sinensis*. The 1285 bp *PsWnt2* was obtained, which included 5′-UTR of 20 bp, 3′-UTR of 188 bp, and an ORF of 1077 bp encoding a polypeptide of 358 amino acids (Figure 1). Sequence analysis predicted the ORF of *PsWnt2* encodes a 24-residue signal peptide (SP), the conserved WNT domain, and 25 conserved cysteine sites were identified in *P. sinensis Wnt2*. The sequence details about *PsWnt2* are shown in Figure 1. 

**Figure 2:**
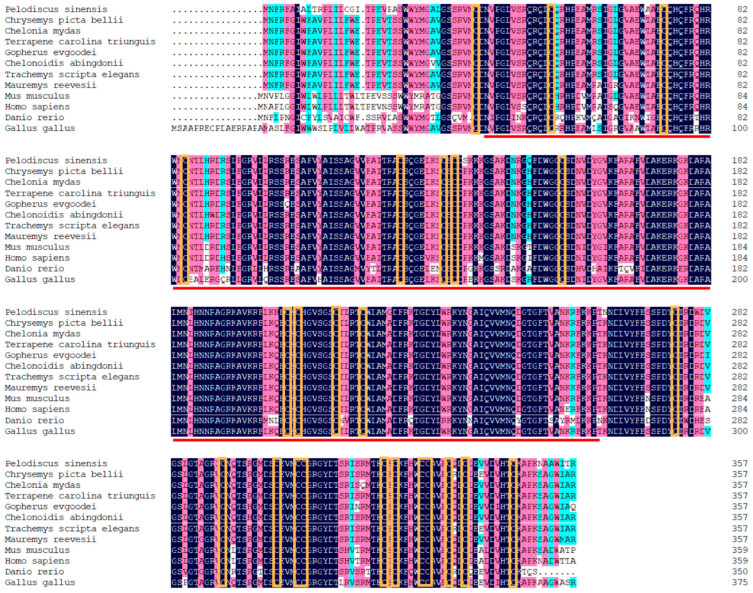
Multiple sequences alignment of *Wnt2* amino acid sequences from different species. Black labels denote amino acids with similarity equal to 100%. The Wnt1 domain of Wnt2 is shown with a red line. The cysteine residues are marked orange.

**Figure 3:**
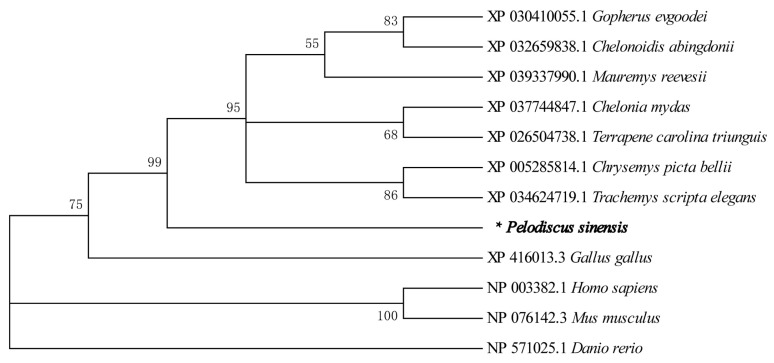
Phylogenetic tree for different species based on *Wnt2* AA sequences. * represents *Pelodiscus sinesis*.

Through the ProtParam program (http://www.expasy.org/tools/protparam.html (accessed on 10 March 2022)), the molecular mass of the deduced *Wnt2* protein was predicted to be 40.338 kDa, and the isoelectric point (pI) was 9.36, which was acidic and uncharged. The hydrophilic coefficient of the *Wnt2* protein was −0.412, and its instability index was 41.66, indicating that the protein was unstable and hydrophobic. Amino acid sequence homology comparison demonstrated that the deduced amino acid sequence of *P. sinensis Wnt2* shared 88.30%, 88.56%, 88.83%, 88.30%, 78.19%, and 63.03% identity with that in the western painted turtle (*Chrysemys picta bellii*), the green turtle (*Chelonia mydas*), three-toed box turtles (*Terrapene carolina triunguis*), the red-eared turtle (*Trachemys scripta elegans*), human (*Homo sapiens*), and zebrafish (*Danio rerio*), respectively (Figure 2, Table S1). The phylogenetic tree based on the *Wnt2* amino acid sequence showed that *P. sinensis* was evolutionarily most closely related to the western painted turtle, the green turtle, three-toed box turtles, and the red-eared turtle, which are clustered into a small branch, then followed by mouse, human, and zebrafish. The phylogenetic analysis showed that *P. sinensis* formed a clade with other turtle species with high similarity (Figure 3). Supplementary Figure S1 showed relative Wnt gene expression during the embryo development stages of Chinese soft-shelled turtle. We found that these Wnt genes have a common domain after comparison (Supplementary Figure S2).

### 3.2. Expression Pattern of PsWnt2 during Embryonic Development

**Figure 4:**
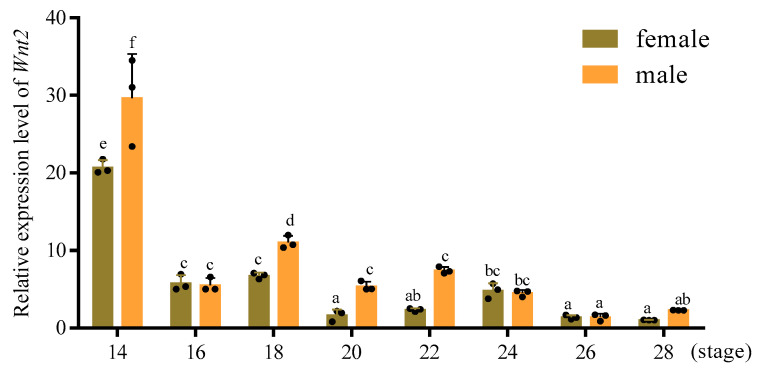
The expression pattern of *Wnt2* in embryonic developmental stages from 14 to 28 of *P. sinensis*. The relative expression level in female gonads at stage 20 was defined as 1. Different letters indicated a significant difference between male and female embryonic gonads at different developmental stages (*p* < 0.05).

Quantitative PCR was used to compare the relative expression levels of *PsWnt2* mRNA in different embryonic developmental stages and types. At the early developmental stage 14 of the male embryos, the expression level of *PsWnt2* was significantly higher than that in female embryos. The *PsWnt2* expression in stage 14 was about 10 times higher than that in stage 20 in male embryos. Moreover, the expression levels of *PsWnt2* at some embryonic stages (14, 18, 20, and 22) in males were significantly higher in females (Figure 4).

### 3.3. Tissue Distribution of PsWnt2 mRNA

**Figure 5:**
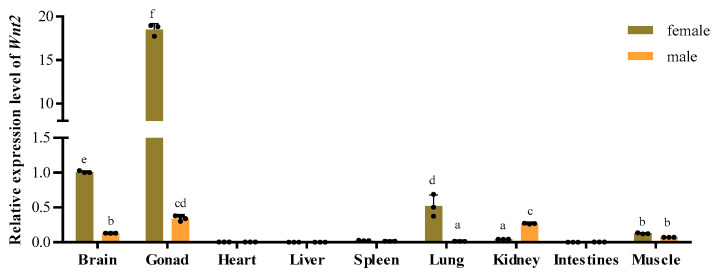
Tissue expression analysis of *Wnt2* in *P. sinensis*. The expression level of *Wnt2* mRNA in the female intestine tissues was the control quantity. Data are shown as means  ±  standard deviations. Various letters indicated statistically significant differences (*p*  <  0.05).

In this study, the expression levels of *PsWnt2* among different tissues were analyzed. We found that it was significantly expressed in the brain, testis, and ovary than in other tissues. In contrast, in males and females, the mRNA expression level of *PsWnt2* had an extremely low expression level in the heart, liver, spleen, and intestine. In addition, *PsWnt2* expression had a female-biased expression pattern in the brain, gonad, and lung. Among all tissues of the two types (female and male), *PsWnt2* expression was highest in the female ovary (*p* < 0.05; Figure 5).

### 3.4. Effects of a Wnt Agonist on P. sinensis Wnt2 Expression

**Figure 6:**
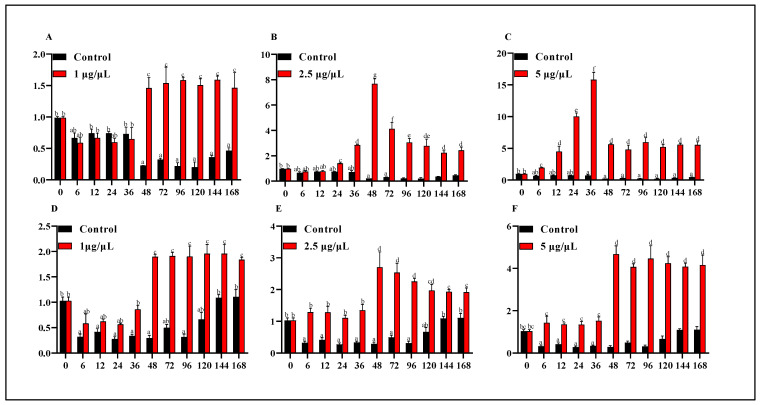
Effects on gene expression of *Wnt2* in different genders of *P. sinensis* embryonic gonads under different concentrations agonists. The vertical axis denoted the expression of *Wnt2* mRNA at each time point. The horizontal axis denoted the experimental time after injecting Wnt agonists. (**A**–**C**) *Wnt2* mRNA level in the female embryos for 1.0 μg/µL, 2.5 μg/µL and 5.0 μg/µL Wnt agonists. (**D**–**F**) *Wnt2* mRNA level in the male embryos for 1.0 μg/µL, 2.5 μg/µL and 5.0 μg/µL Wnt agonists. The data were shown as the mean ± S.D. Various letters indicated significant differences (*p* < 0.05).

After incubation for 15 days, the Chinese soft-shelled turtle embryos were selected to study *PsWnt2* expression in response to the Wnt agonists challenge. This small molecule activates Wnt signaling by inducing catenin via inducing beta-Catenin/TCF transcription. Wnt agonists can significantly influence the expression levels of sex-related genes (Supplementary Figure S3). To investigate the effects of Wnt agonist on the gonads of *P. sinensis*, the expression of *Wnt2* in gonad samples was detected at 11 time points of the experiment using qRT-PCR (Figure 6). In female embryonic gonads, the expression of *Wnt2* in the 1.0 μg/µL treatment group was not significantly different from that in the control group from 6 to 36 h. However, the expression was significantly up-regulated at 48 h and remained high expression level until 168 h. In 5.0 μg/µL and 2.5 μg/µL treatment groups, the peak was reached at 48 h and 36 h, respectively, and then showed a downward trend. In male embryonic gonads, the expression level of *PsWnt2* in three experimental groups was not significantly different at four-time points (6 h, 12 h, 24 h, and 36 h) compared with 0 h. In contrast, *PsWnt2* mRNA expression increased dramatically at 48 h. Moreover, in the 1.0 μg/µL and 5.0 μg/µL Wnt agonist-treated groups, *PsWnt2* expression was maintained at a high expression level after 72 h.

## 4. Discussion

The Wnt signaling pathway is a conserved signaling pathway that plays an important role in regulating and controlling many important internal biological processes. This study showed that the *Wnt2* gene of *P. sinensis* was 1285 bp in length and encoded 358 amino acids, containing a wnt1 domain, a signal peptide, and 25 conserved cysteine sites, which is consistent with the structural characteristics of Wnt-related proteins [21]. Protein sequence analysis revealed that the *P. sinensis Wnt2* shared the highest homology (approximately 80%) with the *Wnt2* of *Chelonia mydas*, *Chelonoidis abingdonii,* and *Chrysemys picta bellii* and a lower identity (approximately 70%) with the *Wnt2* of other vertebrates (*Homo sapiens* and *Gallus gallus*). These findings further demonstrated that the *Wnt2* genes were successfully isolated from the *P. sinensis* in this study. The phylogenetic tree result of *Wnt2* was consistent with the traditional evolutionary relationship, and *Wnt2* was conserved in structure and function.

In this study, the expression levels of *PsWnt2* at some embryonic stages (14, 18, 20, and 22) in male embryos were significantly higher than that in female embryos. This particular expression pattern suggests that the *Wnt2* gene may play a role in regulating gonadal differentiation. Wnt genes have been demonstrated to exert critical roles in embryonic development in vertebrates and invertebrates. Similar results have been observed in *Paracentrotus Lividus*; *Wnt2* mRNA expression has been detected in embryonic developmental stages from the mid-blastula stage to the pluteus larva stage, reaching a peak at the swimming blastula stage [22]. In zebrafish embryos, *Wnt2* is required for early hepatoblast proliferation, and *Wnt2* can interact with *Wnt2b to* participate in swim bladder formation [35]. In addition, *Wnt2* is expressed in gonad formation in drosophila embryos and could promote the development of germ cells. And that, *Wnt2* might stimulate male germ cells to reenter the cell cycle, but not in female embryos [36]. Consequently, *Wnt2* was highly expressed in the early gonadal stage of embryonic development, suggesting that it was involved in determining several specific organ fates in early development.

The *Wnt2* gene is widely expressed during fetal life and functional in organs. For example, *Wnt2* is expressed in the heart and lung and mammary glands of developing mouse embryos [20], suggesting that this gene could regulate the development and differentiation of several tissues. In this study, the *Wnt2* gene had a constitutive expression in diverse tissues of *P. sinensis*, but relative expression levels varied. The high expression level of *Wnt2* in ovaries tissues showed its significance in female gonadal development as in mammals. Conversely, a Wnt2 homolog, Wnt2b, has not been prominently expressed in adult carp’s testis and ovaries [37].

Moreover, studies of *Wnt2* mutant mice showed that it was essential for male fertility as well as that in females [37]. *Wnt2* has been reported to be essential for granule cell growth, and the knockdown of *Wnt2* by siRNA in mice indicates its cell proliferation was inhibited [38]. However, its functional role in determining the gonadal sexual fate among other turtle species still needs further investigation. Interestingly, the *Wnt2* gene was also highly expressed in the brain tissue of the male. A previous report showed that the *Wnt2* gene plays a key role in the later stages of mature brain development [39]. Similarly, *Wnt2* was expressed in the developing brain of the *spider Achaearanea tepidariorum and myriapod Glomeris marginata*, suggesting that *Wnt2* has a possible role in brain regionalization [40]. This observation may have suggested the specific role of *Wnt2* in the brain and gonadal development.

The canonical Wnt signaling pathway was demonstrated to be involved in the morphogenesis of the ovary and testis. Mork and Capel reported that ectopic activation of the Wnt signaling pathway in male gonads results in male-to-female sex reversal in the red-eared slider turtle [41]. On the other hand, suppression of the pathway in female gonads contributed to sex reversal from female to male, indicating that Wnt signaling may be necessary for turtle gonadal differentiation during embryogenesis.

In the Wnt signaling pathway, ligand *Wnt2* specifically regulates the canonical Wnt signaling pathway. The overexpression of *Wnt2* reduced *GSK-3β* transcription and accelerated the accumulation of nuclear level β-catenin [42]. In our study, the *Wnt2* expression of female embryonic gonads in the three treatment groups reached the peak at 48 h or 36 h, respectively, and then the two groups (2.5 μg/µL and 5.0 μg/µL Wnt agonist-treated group) showed a downward trend. In male embryos, the *Wnt2* expression level in three experimental groups increased dramatically at 48 h and remained at a high expression level. Previous studies have shown that Wnt agonist-induced β-catenin accumulation in the nucleus and β-catenin expression was increased with the increase in treatment concentration [43]. We speculated that there might be an exact mechanism that can somehow stabilize β-catenin and rescue cell injury derived from excessive accumulation of β-catenin. On the other hand, *GSK-3β* negatively regulates the Wnt signaling pathway by phosphorylating β-catenin [44]. Therefore, these results suggested that the distinct expression pattern of *Wnt2* may account for the feedback regulation machinery of the Wnt signal pathway response to a Wnt agonist. In contrast, the mechanism might be more sensitive to female embryos than male embryos. In early Ciona embryos, overexpression of β-catenin could directly change the endoderm cell differentiation’s fate [45].

Taken together, the increased expression of *Wnt2* may activate the Wnt signaling pathway, stimulate the activation of Wnt target gene expression, and influence the fate of female gonads differentiation. As the interaction between the Wnt signaling pathway, gonadal development, and sex determination in turtles is a complex process, the information on the Wnt signaling molecule regulating this mechanism is limited to very few studies. Further studies are envisaged to elucidate the specific role of the requirement of Wnt/β-catenin signaling in the turtles by the experiments of the RNA interference of the *Wnt2* gene in the gonads and bring a new perspective to the precise requirements for Wnt/β-catenin signaling in early gonadal development patterning.

## Figures and Tables

**Table 1:** The information of the primers for *Wnt2* amplification in *P. sinensis*.

Primer Name	Primer Sequence (5′–3′)	Application
UPM	CTAATACGACTCACTATAGGGCAAGCAGTGGTATCAACGCAGAGT	5′ and 3′ RACE PCR
UPM short	CTAATACGACTCACTATAGGGC	
*Wnt2*-F	CGGAGTGAAGTGTTTCTAATATGAA	CDS clone
*Wnt2*-R	ACAGCCTTCCTTCCCGCTCT	
*Wnt2*-F2	TCACAAGGGCATGTAGTCAAGGGGA	
*Wnt2*-R2	GTGTACGTCCACCACCTCCAGGCAG	
*Wnt2*-3′-GSP	CTGTATCAGAGACTGGGATGTAGGCT	3′ RACE
*Wnt2*-qF	CAAGACGGCACTGGTTTCAC	qPCR
*Wnt2*-qR	GTCCCAAGGGAGCCTACATC	
*Sox3*-F	GAGTGTAGAGGTGGAATGGAAACG	
*Sox3*-R	AAACCCTCAAGCAGGATACGG	
*Dmrt1*-F	CCGCCTCGGGAAAGAAGTC	
*Dmrt1*-R	TGCTGGATGCCGTAGTTGC	
*Wnt4*-F	GAGGTGATGGACTCGGTGCG	
*Wnt4*-R	CCCGTTCTTGAGGTCGTGGTC	
*Amh*-F	CGGCTACTCCTCCCACACG	
*Amh*-R	CCTGGCTGGAGTATTTGACGG	
18S rRNA-F	AAAGGAATTGACGGAAGGGCAC	Internal control
18S rRNA-R	GCTCCACCAACTAAGAACGG	
Ps4085-F	GTTTGAAGTGCTGCTGGGAAG	Sex identification
Ps4085-R	TTCCCCGTATAAAGCCAGGG	
COI-F	CAACCAACCACAAAGACATTGGCAC	
COI-R	ACCTCAGGGTGTCCGAAAATCAAA	

## Data Availability

The data that support the findings of this study are available from the corresponding author upon reasonable request.

## Supplementary Materials

The following supporting information can be downloaded at: https://www.mdpi.com/article/10.3390/life13010188/s1, Figure S1: Relative *Wnt* genes expression during embryo development stages; Figure S2: The structure of *Wnt* proteins in *Pelodiscus sinesis*; Figure S3: Expressions of sex-related genes after treatment *Wnt* agonist; Table S1: Comparison of deduce amino region of *P. sinensis Wnt2* with that of other species.
