# Supplementary material for: Identification and Expression Analysis of Wnt2 Gene in the Sex Differentiation of the Chinese Soft-Shelled Turtle (Pelodiscus sinensis)
Source: Life (Basel). 2023 Jan 9;13(1):188. doi: 10.3390/life13010188 (PMC9864750; doi:10.3390/life13010188)
Supplement: Supplementary file 1 [file life-13-00188-s001.zip › life-2079020-supplementary.pdf]

Supplementary materials

Figure S1. Relative Wnt genes expression during embryo development stages.

A (females) , B (males)

Figure S2. The structure of Wnt proteins in pelodiscus sinensis.

The green boxes indicate the conserved Wnt1 domains. The blue boxes indicate transmembrane regions. The red boxes indicate signal peptides.

Figure S3. Expressions of sex-related genes after treatment Wnt agonist.

(A-D) Expressions of *Dmrt1*, *Amh*, *Sox3*, and *Wnt4* respectively in females after treatment with Wnt agonist. (E-H) Expressions of *Dmrt1*, *Amh*, *Sox3*, and *Wnt4* respectively in males after treatment with Wnt agonist.

Table S1. Comparison of deduce amino region of P. sinensis Wnt2 with that of other species.

Figure S1

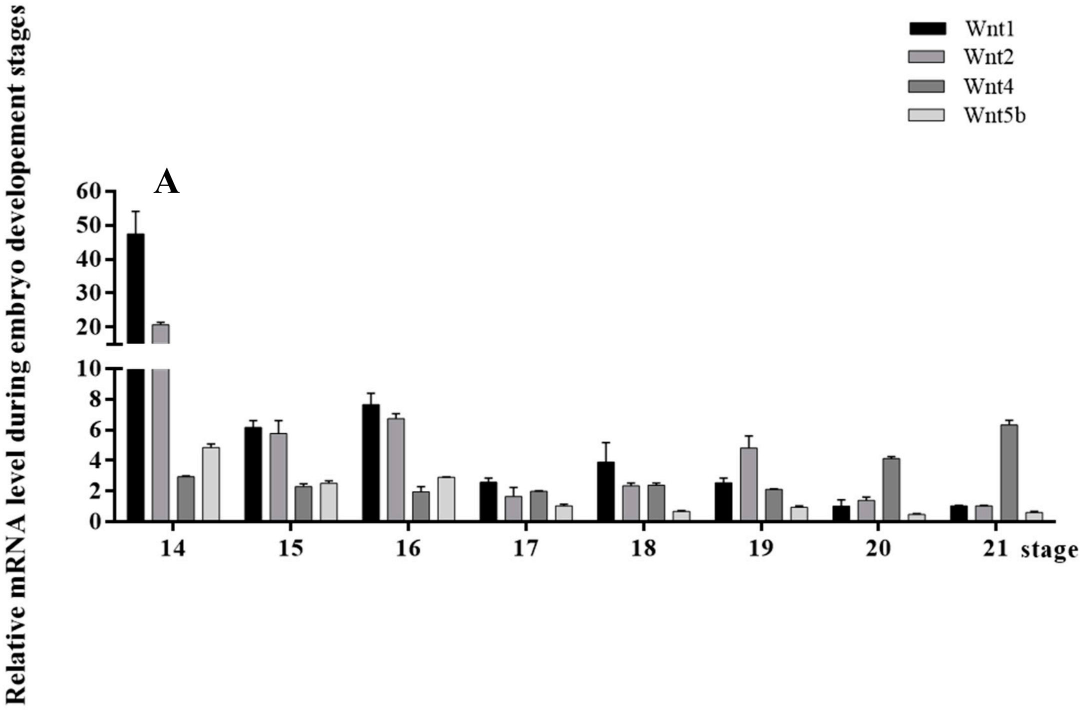
Wnt1
Wnt2
Wnt4
Wnt5b
A
Relative mRNA level during embryo developement stages
60
50
40
30
20
10
8
6
4
2
0
14
15
16
17
18
19
20
21
stage

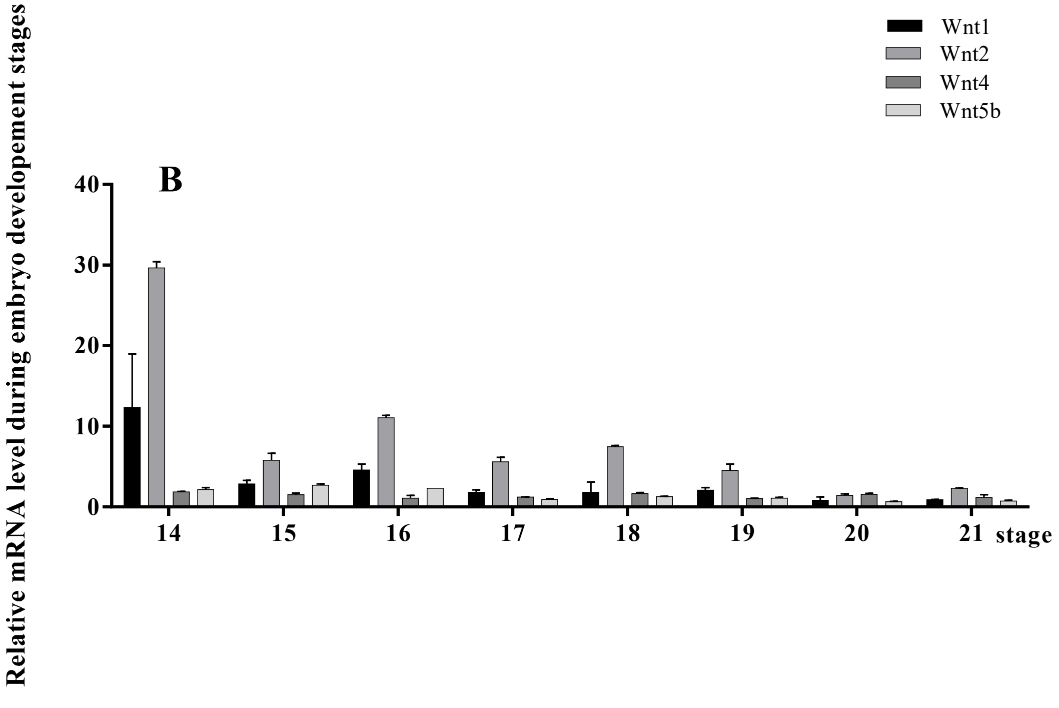
Wnt1
Wnt2
Wnt4
Wnt5b
B
Relative mRNA level during embryo developement stages
40
30
20
10
0
14
15
16
17
18
19
20
21
stage

Figure S2

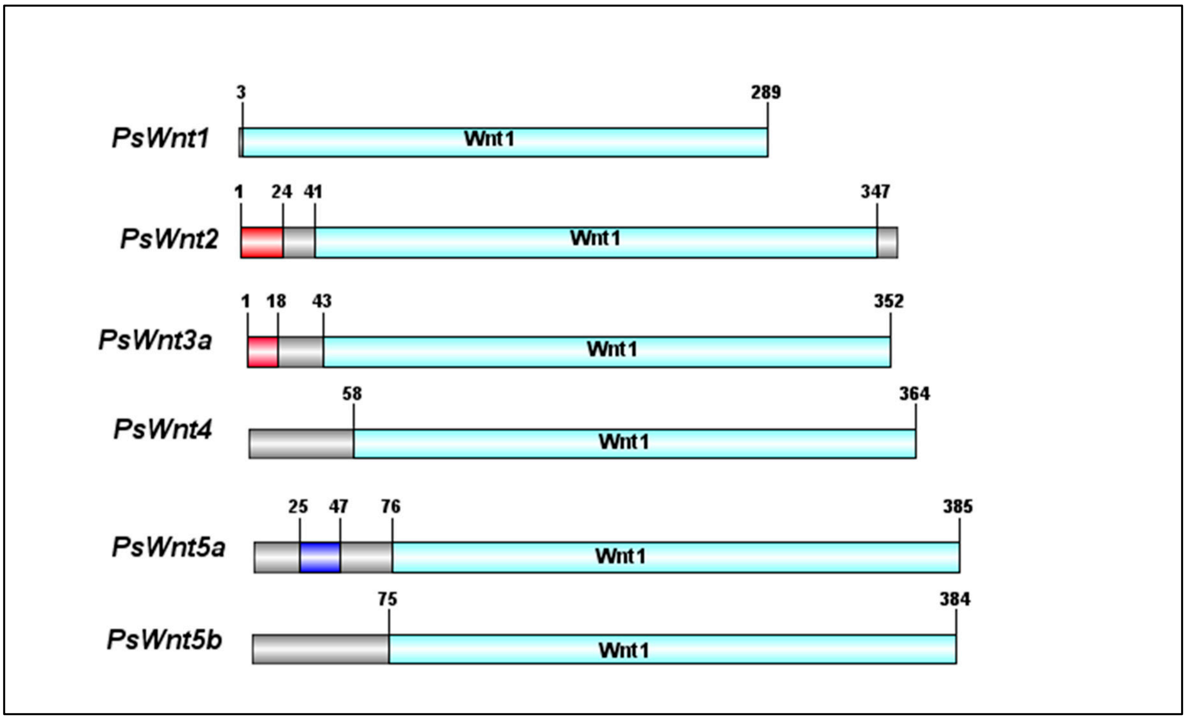
PsWnt1
3
289
Wnt1
PsWnt2
1
24
41
347
Wnt1
PsWnt3a
1
18
43
352
Wnt1
PsWnt4
58
364
Wnt1
PsWnt5a
25
47
76
385
Wnt1
PsWnt5b
75
384
Wnt1

FigureS3

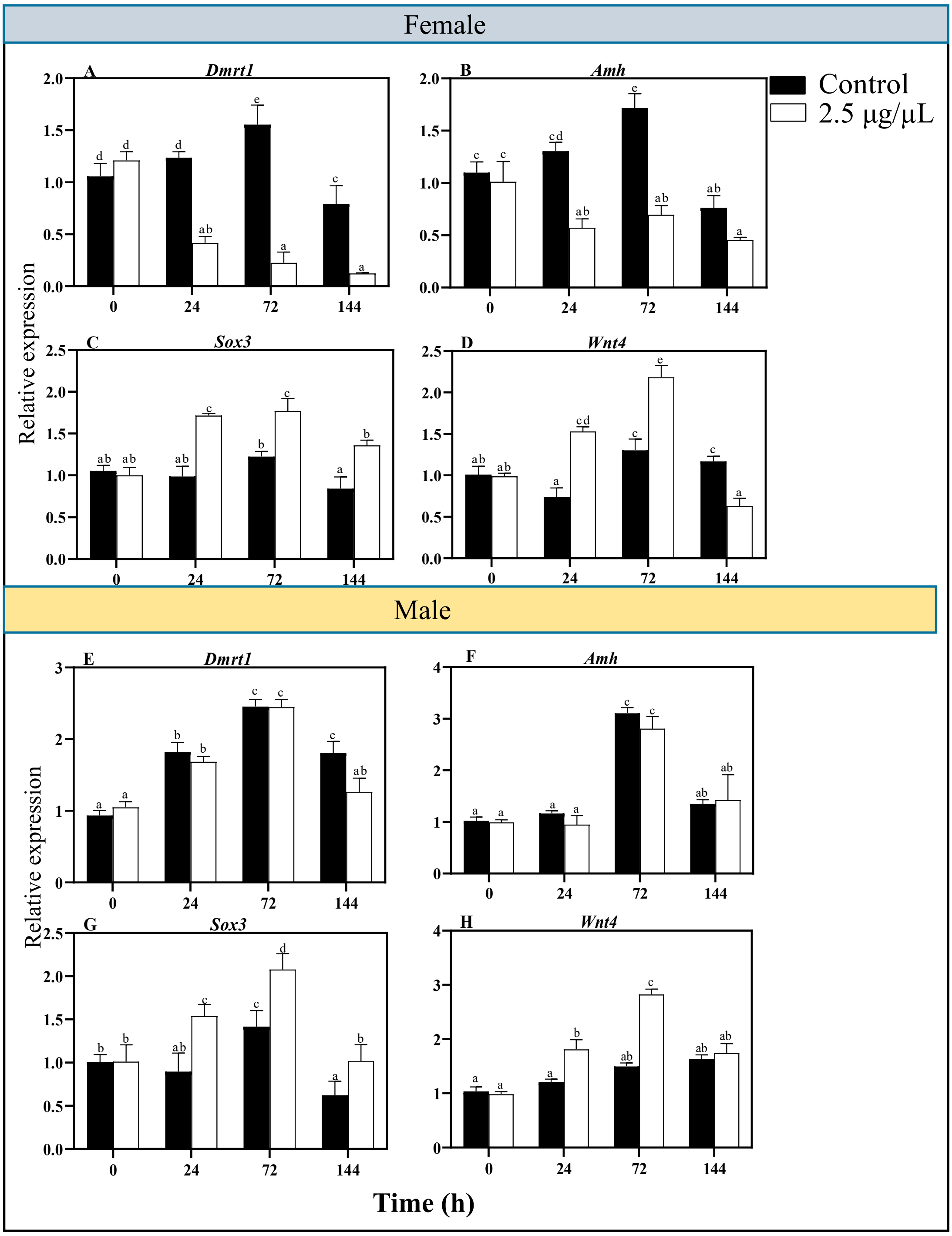

Female
Male
Control
2.5 μg/μL
A
Dmrt1
B
Amh
C
Sox3
D
Wnt4
E
Dmrt1
F
Amh
G
Sox3
H
Wnt4
Relative expression
Relative expression
Time (h)

Table S1.

Species	Identity
Chrysemys_picta_bellii	88.3%
Chelonia_mydas	88.56%
Terrapene_carolina_triunguis	88.83%
Gopherus_evgoodei	86.79%
Chelonoidis_abingdonii	87.77%
Trachemys_scripta_elegans	88.30%
Mauremys_reevesii	87.50%
Mus_musculus	79.79%
Homo_sapiens	78.19%
Danio_rerio	63.03%
Gallus_gallus	80.05%
